# Making clinical trials more relevant: improving and validating the PRECIS tool for matching trial design decisions to trial purpose

**DOI:** 10.1186/1745-6215-14-115

**Published:** 2013-04-27

**Authors:** Kirsty Loudon, Merrick Zwarenstein, Frank Sullivan, Peter Donnan, Shaun Treweek

**Affiliations:** 1 Division of Population Health Sciences, University of Dundee, Kirsty Semple Way, Dundee DD2 4BF, UK; 2 Department of Family Medicine Director, Centre for Studies in Family Medicine, Schulich School of Medicine & Dentistry Western University, 245-100 Collip Circle, Research Park, London, ON N6G 4X8, Canada; 3 Health Services Research Unit, University of Aberdeen, 3rd Floor, Health Sciences Building, Aberdeen, Scotland, UK

**Keywords:** Pragmatic, Explanatory, Clinical trials, Trial design, Applicability

## Abstract

**Background:**

If you want to know which of two or more healthcare interventions is most effective, the randomised controlled trial is the design of choice. Randomisation, however, does not itself promote the applicability of the results to situations other than the one in which the trial was done. A tool published in 2009, PRECIS (PRagmatic Explanatory Continuum Indicator Summaries) aimed to help trialists design trials that produced results matched to the aim of the trial, be that supporting clinical decision-making, or increasing knowledge of how an intervention works. Though generally positive, groups evaluating the tool have also found weaknesses, mainly that its inter-rater reliability is not clear, that it needs a scoring system and that some new domains might be needed. The aim of the study is to: Produce an improved and validated version of the PRECIS tool. Use this tool to compare the internal validity of, and effect estimates from, a set of explanatory and pragmatic trials matched by intervention.

**Methods:**

The study has four phases. Phase 1 involves brainstorming and a two-round Delphi survey of authors who cited PRECIS. In Phase 2, the Delphi results will then be discussed and alternative versions of PRECIS-2 developed and user-tested by experienced trialists. Phase 3 will evaluate the validity and reliability of the most promising PRECIS-2 candidate using a sample of 15 to 20 trials rated by 15 international trialists. We will assess inter-rater reliability, and raters’ subjective global ratings of pragmatism compared to PRECIS-2 to assess convergent and face validity. Phase 4, to determine if pragmatic trials sacrifice internal validity in order to achieve applicability, will compare the internal validity and effect estimates of matched explanatory and pragmatic trials of the same intervention, condition and participants. Effect sizes for the trials will then be compared in a meta-regression. The Cochrane Risk of Bias scores will be compared with the PRECIS-2 scores of pragmatism.

**Discussion:**

We have concrete suggestions for improving PRECIS and a growing list of enthusiastic individuals interested in contributing to this work. By early 2014 we expect to have a validated PRECIS-2.

## Background

Pragmatic trial design hit the research headlines in September 2011 over the discussion of the Advisory Committee to the US Food and Drug Administration (FDA) to approve rivaroxaban to minimise stroke risk for patients with atrial fibrillation [1]. This decision to recommend rivaroxaban, over the more well-known alternative anticoagulant treatment warfarin, was not unanimous; debate arose over the pragmatic study design used in the trial and the applicability of the trial results. The majority (9 *vs.* 2, one abstention) thought the pragmatic design of the ROCKET AF trial was good but some questioned the rigor of the design and the compliance rates seen in the trial. The debate highlighted the impact design decisions have on clinicians’ and others’ confidence in trial results and, in particular, the need to select patients who are ‘truly reflective of the type of patients physicians would see in everyday practice’ [1].

 In the UK, the National Institute for Health and Clinical Excellence (NICE) is often embroiled in debate over decisions for recommending or withholding treatments if uncertainties over a treatment’s effects. If NICE recommend ‘Only in Research’ (that is, treatments that are not proven to have an effect should only be administered to patients in the NHS through research,) following Health Technology Appraisals, this is often taken as a ‘No’ to a new treatment [2]. Hence the role of pragmatic randomised controlled trials (RCTs) in comparative effectiveness research is of increasing interest in producing evidence for ‘real world’ benefits and risks [3].

Pragmatic trials were first proposed by Schwartz and Lellouch in 1967 as trials performed under normal conditions with the intention of providing results that are more applicable to clinical practice and decision-making. The alternative, taking a more explanatory approach, leads to tightly controlled trials under ideal conditions that aim to provide understanding of how treatments work [4]. Explanatory trials have an important role but healthcare interventions are seldom given under circumstances similar to those used in such trials [5,6]. Many authors have highlighted the need for trials with greater applicability [7-9]; in other words, trials that pay attention to external validity as well as internal validity. Lack of consideration of external validity is the most frequent criticism by clinicians of RCTs, systematic reviews and guidelines [7].

**Figure 1:**
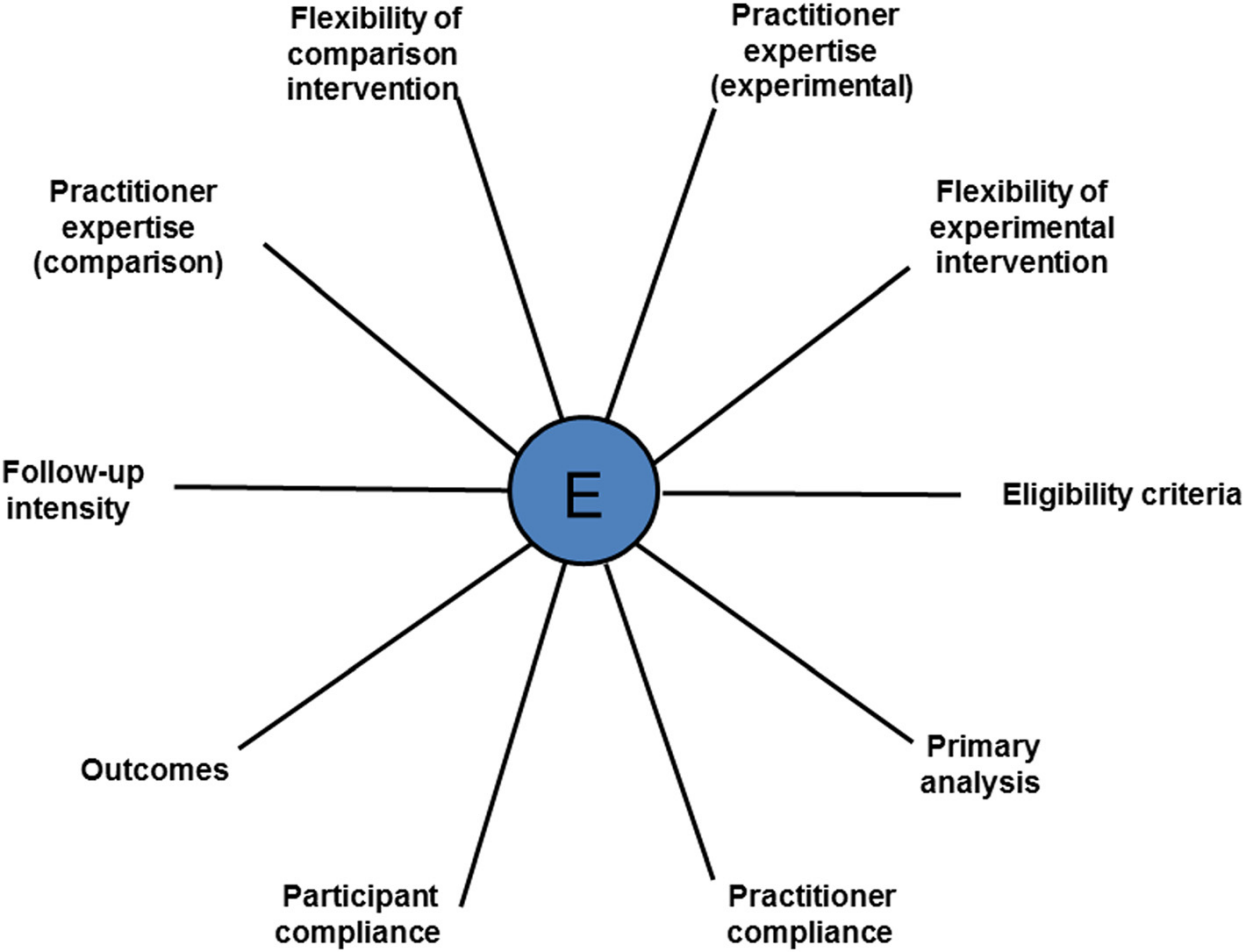
**Pragmatic Explanatory Continuum Indicator Summary (PRECIS) **[10]**.**

But how does a trialist know that his or her trial has the right design? In 2009 a tool called the Pragmatic Explanatory Continuum Index Summary (PRECIS) was published simultaneously in two journals to help trialists think more carefully about the impact their design decisions would have on applicability [10,11]. PRECIS is a 10-spoked ‘wheel’ (see Figure 1 and Additional file 1) with 10 domains based on trial design decisions (for example, participant eligibility criteria, practitioner expertise and choice of comparator). Trials that take an explanatory approach produce wheels nearer the hub; those with a pragmatic approach are closer to the rim.

Our hypothesis is that pragmatic trials do not compromise internal validity (we should not mistake realism for bias) and that explanatory trials overestimate effect size compared to more pragmatic designs and that recommendations regarding trial design, especially for regulatory trials, need to be modified. A modified PRECIS tool is, however, needed first to be able to separate explanatory and pragmatic trials in a consistent way.

### Prior research rationale

PRECIS was developed by more than 25 international trialists and methodologists, including ST and MZ, working between 2005 and 2008 [12], linked to simultaneous work on the CONSORT extension for pragmatic trials [13]. The aim was to produce a tool that helped trialists match their design decisions to the purpose of the trial, be that informing a clinical decision or increasing knowledge of how an intervention works. Currently, PRECIS provides a simple graphical summary of 10 key design decisions (see Figure 1) to assist trial designers in ensuring their decisions are consistent with the purpose of the trial.

PRECIS has never been formally validated but a diverse group of methodologists and trialists have used the tool; it is interesting to note who has used the PRECIS, as it was developed for all trialists to make design decisions [10]. Eight groups have studied PRECIS [14-21] and these studies have all concluded that PRECIS was useful in designing trials, or to assess how pragmatic or explanatory trials included in systematic reviews were. All suggested that PRECIS needed some modifications.

### PRECIS studies

In the study by Riddle [14] planning total knee surgery the trial investigators came from a wide range of specialties (physical therapy, biostatistics, rehabilitation medicine, psychiatric and behavioural sciences, medicine, rheumatology). Riddle [14] found the PRECIS tool useful for discussing trial design and achieving consensus during a 1-day face-to-face meeting. To enable constructive discussion Riddle [14] used a PRECIS wheel with spokes that were 0 to 4 cm in length with the zero point at the hub (explanatory) and 4 cm at the rim of the wheel (pragmatic). They used the tool as it was intended - to design the protocol for a clinical trial; pre-meeting, ideal score and post-consensus meetings when all the raters were present but blinded to each other’s score. Raters marked a point on the wheel which was then measured. This must have been a little time-consuming but allowed the participants to quantify disagreement between raters which could then be discussed. Variation in scoring for the 10 PRECIS categories, as reflected in average standard deviation moved from initial assessment of 0.83, to 1.16 for the ideal trial for individual raters, to 0.61 for the final assessment.

In his first paper using PRECIS, Bratton [18] scored three trials on tuberculosis treatment with another researcher. They combined the spokes on expertise and inserted a domain for ‘blinding’ and found the tool helpful to pinpoint weaknesses in the trial design as well as assisting trialists to consider the applicability of their trials. Bratton [21] then used PRECIS again in an ongoing trial of an autoimmune disease non-inferiority trial; the degree of pragmatism was considered by a trial manager, two clinicians with expertise in the trial area of pemphigoid and two statisticians. Bratton [21] advocated the procedure described by Riddle [14] and so his group also used a blank PRECIS wheel approximately 15 cm in diameter. While they recommended using PRECIS to achieve consensus they had a few concerns that there was a great deal of variability scoring domains depending on expertise of raters and interpretation of guidance from the original Thorpe paper [10,11].

In the study by Tosh *et al*. [15], three doctors who had research backgrounds and specialised in psychiatry, used PRECIS to evaluate mental health protocols. Tosh’s [15] group decided they wanted to quantify how pragmatic 10 protocols were, and decided to score PRECIS using 1 (most explanatory) to 5 (most pragmatic) with zero for domains that did not contain any information. They used 0 to 30 for an explanatory trial, 31 to 39 for a trial balanced between pragmatic and explanatory, and 40 to 50 for a pragmatic trial. Calling their modified PRECIS, the Pragmascope tool, reliability was reasonable for the raters (weighted Kappa = 0.72).

Koppenaal *et al*. [17] adapted PRECIS - PRECIS-Review tool (PR-tool) - to rate trials in a systematic review to determine which intervention would improve lifestyle most in general practice. As the original PRECIS tool produces a wheel like figure for each trial with no scoring system, it is hard to assess how applicable to routine care a systematic review is. So, Koppenaal *et al*. [17] proposed scoring the domains and each trial using a scoring system of 1 to 5 as well as 0 to 100%; they found that a scale of 1 to 5 was less subjective compared with a 0 to 10 scale. By scoring all the trials in the systematic review they were able to determine which trial was most pragmatic and overall how pragmatic and thus how applicable the review was. They were also able to determine how heterogeneous the individual trials were. They did have concerns though about arbitrary scoring and that more pragmatic scoring occurred when there was inadequate information. Koppenaal *et al*. [17] proposed that two researchers should score RCTs to improve validity. His team also highlighted problems with context that the PR-tool did not pick up and that weighting of domains may be dependent on the situation. Overall he concluded that the PR-tool-PRECIS with a scoring system was very helpful to users of RCTs and systematic reviews.

Glasgow *et al*. [16] invited nine raters who were medical doctors or had doctorates and were experienced researchers to use PRECIS; six were involved in the trials they were rating, Practice-based Opportunities for Weight Reduction (POWER) and three independent raters who had nothing to do with the trials being scored. Glasgow *et al*. [16] used a score from 0 (most pragmatic) to 4 (most explanatory) but the researchers required several conference calls to discuss the domains and scoring. There were problems with inter-rater reliability and raters determining that their own trial was more pragmatic than independent raters; Glasgow questioned if this was due to bias or more trial knowledge.

Witt *et al*. [19] used PRECIS to evaluate acupuncture trials for a systematic review. Her team of PhD and MD raters had more than 10 years of experience in clinical research and had worked on aspects of research methodology, with experience in acupuncture. Witt *et al*. [19] also chose to use a score, 1 to 5, to allow score comparisons of inter-rater correlations and ensuring results could be presented as figures not just diagrams. Witt’s groups observed that the first round of scorings were highly heterogeneous but that inter-rater reliability improved following discussion of domains.

Selby *et al*. [20] for a trial on smoking cessation had a team of six raters with a range of expertise (one academic family physician with an interest in smoking cessation; one cardiac rehabilitation physician with expertise in pharmacoeconomics; one addiction medicine physician and clinical scientist with a focus on tobacco dependence; one pharmacist with expertise in pharmacoeconomics; one pharmacologist with clinical research and medical affairs experience in the pharmaceutical industry; and one consultant physician with pharmacoeconomics and policy advice experience in Quebec). Selby *et al*. [20] highlighted how difficult it was to use a Visual Analogue Scale for raters working online using PRECIS to guide the protocol development of a smoking cessation trial. As the printers they were using were all different, so too hard to use a VAS for comparison purposes, they chose to use a 20-point scale, where 1 represented ‘entirely explanatory’ and 20 represented ‘entirely pragmatic’ making it easier to score using email. They scored the protocol twice using a modified Delphi process. Selby advocates that using a system of scoring PRECIS anonymously allows different views to be expressed and assists in consensus decision-making from multidisciplinary raters. This ensures the best design to answer the study question. Rater scores varied less after the second round than the first indicating convergence in opinions following discussion.

To summarise, all published users of PRECIS have used either Visual Analogue Scales or Likert scoring systems to enable individual raters to compare decisions and measure how pragmatic or explanatory a domain is. Most groups have also used PRECIS for consensus decision-making. A summary of their key findings is in Table 1.

**Table 1:** Summary of existing work with PRECIS

**Reference**	**Scale**	**RCTs**	**Protocols**	**Raters (*n*)**	**Consensus scoring**	**Comments**
Riddle (2010) Pain coping skills training for patients - knee arthroscopy	0-4 cm circle VAS	X	1	7	YES (individual scoring then consensus score)	Useful to focus trial design discussion. PRECIS scoring: Initial, personal ideal and then post meeting. 1-day face-to-face meeting to discuss trial. PRECIS facilitates discussion.
						Trial design changed: domain ‘practitioner expertise’ - needed to do psychologist training and domain ‘practitioner adherence’ - rigorous ongoing assessment to check intervention as intended. Visual analogue scale cannot be used if ‘online ratings’.
Glasgow (2010) Weight loss in obese patients with comorbid conditions	0-4 scale	3	X	9	NO	PRECIS improved ‘transparency’ in trial design decisions, encouraged others to use.
						Domain most variation: Primary analysis.
						Trialists rate own trial more pragmatic than other raters. Not clear if original criteria are sufficient to provide a comprehensive profile.
Tosh (2011) Mental health	1-5	X	10	3	NO	‘Useful tool’. Cumulative scores for all 10 PRECIS domains. Experimental 0–15, Pragmatic >35, 31–19 interim where trial balances pragmatic and explanatory domains. Scoring depends on rater’s perspective. 0 for missing information
Koppenhaal (2011) Systematic review on lifestyle improvements in General Practice	1-5%	20	X	2	YES (individual scoring then consensus score)	‘Useful estimate by estimating quantitatively how pragmatic each RCT is’. Chose PRECIS as explanatory/pragmatic continuum and visual analogue scale. Domains most variation: Practitioner expertise (comparison), primary analysis. Tried 1–10 score but too much difference between consecutive scores not meaningful, still concerned arbitrary - important to reduce subjectivity so two raters (third rater if not consensus). Weighting could be important; eligibility criteria important but flexibility of the comparison intervention may be less important. Problems using PRECIS due to reporting as CONSORT guidelines not being followed.
Bratton (2011)	No scoring	3	X	2	Joint discussion	Blinding inserted and combined experience of practitioner expertise for comparison and experimental intervention as postulated no difference in expertise in these trials.
Bratton (2012)		1 (ongoing)	X	6	NO	‘Useful tool for designing, conducting and reporting trials’. Strongest consensus ‘flexibility of the comparison intervention’ and ‘practitioner adherence’ domains. Most disagreement on ‘eligibility criteria’ and ‘participant compliance’.
Witt (2012)	1-5	10	X	5	YES (individual scoring then consensus score)	“PRECIS useful but needs further development”. CONSORT guidelines for reporting pragmatic trials should be expanded. Recognised that PRECIS originally intended for trial design but useful tool for appraising published RCTs for systematic reviews.
Selby (2012)	1-20	X	1	6	YES (individual scoring then consensus score)	PRECIS useful to help interdisciplinary co-investigators rate their study design. Used two rounds of Modified Delphi process used to reach consensus. 20-point numerical scale approximated a continuous scale allowing ‘easier, more accurate and more stable coding of the response using e-mail’ - extreme anchor points 1–20, discouraged rating the domains beyond the numbers provided.

### Study objectives

1. To produce an improved and validated version of the PRECIS tool.

2. To use this tool to compare the internal validity of, and estimate effect from, a set of explanatory and pragmatic trials matched by intervention.

## Methods

### Study design

There will be four phases to the study.

•Phase 1: We will develop PRECIS-2 through expert consensus and conduct a two-round electronic modified Delphi survey.

•Phase 2: Alternative PRECIS-2 models will be user-tested in Dundee, Scotland and London, Ontario, Canada using one-to-one testing or small group testing.

•Phase 3: Validity and reliability testing of PRECIS-2 using a sample of 15 to 20 trials and a minimum of 15 trialists/methodologists from around the world.

•Phase 4: Comparing the internal validity and effect estimates of matched explanatory and pragmatic trials

### General and phase 1

First, we will establish a steering group (PD, KL, ST, FS, MZ). Second, we will design the first round of a modified Delphi consensus method [22] to discuss improving the current PRECIS tool and create PRECIS-2. We will generate a web-based questionnaire using the SurveyMonkey® survey tool ‘select’ version (that is, not the free version).

It is widely accepted that the initial round of a modified Delphi process can be a structured questionnaire if there has been a systematic review of the literature and there is adequate information to base the survey on [23,24]. Our questionnaire will be based on problems that have been published by users of PRECIS and the report from a brainstorming meeting at the University of Dundee on 8 June 2012. Topics to be discussed in the survey will include Scoring, Domains and Design. There will be an introduction to the survey and all participants will be advised that their assistance in participation in the survey will be acknowledged in any future publication. We will be careful in designing the questionnaire not to lead respondents into expected answers but keep questions open to gain maximum information, allowing us to develop a full range of PRECIS alternative models for user testing and ‘explore or expose underlying assumptions’ [25].

The survey will take around 10 minutes. It will not be compulsory for participants to answer any of the questions. All participants will have an opportunity to leave comments as well as multiple choice yes or no answers. Responses from individuals will be known only to the moderator (KL), so participant anonymity and confidentiality of responses will be ensured. Reasons for declining participation will be collated if solicited.

We will pilot test the SurveyMonkey® questionnaire with the PRECIS steering group and the four participants involved in the initial Dundee brainstorming meeting (June 2012) until the steering group are satisfied with the Survey. We will invite (by email using MailMerge) all those who have cited PRECIS from the Thorpe 2009 [10,11] publications and have not previously been involved in the development of PRECIS to participate in in all four phases and start by completing SurveyMonkey® questionnaire to give advice to improve PRECIS. We will ask them to pass on to interested colleagues or let us know if they know of anybody who may be interested in participating.

We will send out reminders after 2 weeks, and another reminder to those who had initially said they were interested but who had not completed the survey after a further 2 weeks to optimise recruitment [26]. We will take note of the response rate for the first round, pre and post reminder before moving onto the second round. Following the first round of the Delphi, the steering group will review the responses and select the topics to go forward to the next round. A report will be prepared for all participants based on Round 1 responses and a link for a further Delphi round using SurveyMonkey® will be sent to everyone who has stated their interest (pilot tested as described before). A further reminder will be sent for the Delphi Round 2 participants to encourage maximum participation and response rates will be noted. A report will be prepared for all Round 2 participants. Through brainstorming, the Steering group will use the results of the second round of the Delphi, including free-text responses, to develop PRECIS-2 models. Additional input may be sought from the Dundee brainstorming group in finalising models for Phase 2 user testing.

### Phase 2

A review of the first part of Phase 1 will be given as an introduction at meetings in Dundee, Scotland and London, Canada which will be attended by participants gathered for user testing of different PRECIS models. This will be on a one-to-one basis or in small groups. User-testing [27] involves, a user (for example, a trialist) being presented with the tool being tested (for example, PRECIS-2) and a topic list of questions and tasks involving a specific trial that makes use of the tool. The key advantages of user-testing are that it provides feedback from the user’s perspective and is quick; testing generally takes no more than 1 hour. We will also use a brief training package at the start of the meeting. To encourage maximum participation from all participants, the user testing group size will be small. Ideally this will be six people maximum but logistically this may not always be possible. Discussion on the different PRECIS models will be facilitated by KL, assisted by MZ or ST. One-to-one user testing will be carried out by KL or ST with the other acting as the assistant. With permission of participants, all meetings will be audio-taped and then transcribed. If the participant does not give permission then only notes will be taken during user testing of PRECIS models.

Models for user testing will pick up on themes highlighted in Phase 1, in particular scoring (if at all), weighting, domains and design. We will make sure that we include head-to-head comparisons between the old and the new PRECIS tools. The topic guide with questions for the user will be piloted by the Dundee Brainstorming participants and the Steering group prior to Phase 2 user testing commences.

The project team will review notes and transcriptions together after each test, looking for barriers and facilitators to the use of PRECIS-2, categorised according to the severity of the problem: high (critical errors such as incorrect interpretation or high degree of uncertainty or dissatisfaction), medium (much frustration or unnecessarily slow use), and low (minor or cosmetic problems). By modifying PRECIS-2 in response to user-testing, and then testing the new version in further user-testing, the tool will be much more likely to be relevant and useful to its intended user group of trialists and methodologists. The version of PRECIS-2 that finally emerges from iterative user-testing will be put into Phase 3.

### Phase 3: validity and reliability testing

Unlike with the development of the original PRECIS, we intend to formally test PRECIS-2’s validity and reliability at the development stage. To be feasible, and to ensure that a diverse range of trialists and methodologists can be involved, we will use an electronic process for this work.

The electronic process will involve a minimum of 15 trialists/methodologists from around the world considering a mixed sample of trial reports (chosen by the Steering Group during Phase 1 and 2) using PRECIS-2. All participants will be sent a concise PRECIS-2 training package and between 10 and 15 trials (See Sample size and statistics). Some will be extremely pragmatic in design, some extremely explanatory, with the other trials a mixture. These trials will be selected to exemplify the different trial types and demonstrate particular aspects of trials that would stimulate discussion to develop PRECIS-2. Some will be drug trials. Participants will be asked to give subjective global ratings of pragmatism before being asked to use PRECIS-2. If necessary we will modify PRECIS-2 and repeat the exercise with trialists and methodologists if validity and reliability are initially poor. Rating a trial using PRECIS takes between 10 and 20 minutes and we expect PRECIS-2 to be similar. There will be a financial payment to each trialist/methodologist completing the PRECIS-2 ratings of approximately £200.

### Phase 4: Comparing the internal validity and effect estimates of explanatory and pragmatic trials

By the end of Phase 3 we will have a validated tool (PRECIS-2) for judging how explanatory or pragmatic a clinical trial is. Phase 4 will use this tool to answer speculation [28,29] that pragmatic trials sacrifice internal validity in order to achieve applicability.

We will use the PRECIS-2 tool to create a database of around 200 trials that take a pragmatic approach to design, are rated for pragmatism (using PRECIS-2) and risk of bias and which collate information on effect size. We have developed and tested an initial search strategy for this search:

1. ‘pragmatic trials’

2. ‘management trials’

3. ‘efficiency trials’

4. ‘practical trials’

5. ‘effectiveness trials’

6. ‘efficacy trials’

7. ‘field trials’

8. ((‘real-world’ OR ‘real life’) AND trials OR trial))

9. MH ‘Clinical Trials as Publication Type’

10. S1 or S2 or S3 or S4 or S5 or S6 or S7 or S8

11. S9 AND S10

This search is being refined at the time of writing and will be in a definitive state by the start of Phase 4. We will extract the following information from all retrieved trials: topic of trial, number of centres, sample size, randomization, allocation and blinding methods, type of statistical analyses, participant characteristics (for example, age, condition, baseline severity and so on), duration of trial, and effect sizes and measures of variance for the primary outcome. The degree of pragmatism will be assessed using the PRECIS-2 tool and the risk of bias assessed using the Cochrane Risk of Bias Tool [30]. The database will be made publicly available as a searchable and extendable (that is, new studies can be added) web resource. In addition to the database, we will make PRECIS-2 training and other guidance, a summary of other PRECIS literature and other tools to support the design of trials available via the website.

Next we will match some of the pragmatic trials with more explanatory trials of the same intervention, condition and participants. It is impossible to say which interventions we will match without having created the database of pragmatic trials but it is almost certain that all the interventions we will use for matching will be drugs. We will extract the same information for these matched trials as we extracted for the more pragmatic trials. Effect sizes for the trials will then be compared in a meta-regression (see Sample size and statistics).

### Participants

We will involve an international group of methodologists and trialists, healthcare professionals, and other individuals with trial expertise, in particular researchers who have stated an interest in the project and said that they would like to be involved in the validation of PRECIS-2. This will include those involved in the original PRECIS tool development.

### Identifying participants

We will initially identify participants to assist in PRECIS development, starting with the SurveyMonkey® questionnaire, from all those who cited the original Thorpe *et al*. articles [10,11] via email invitation. Participants in additional phases will be those who stated they wanted to take part in the Delphi survey, and trialists and methodologists known to the PRECIS steering group through collaborations including the Cochrane Methodology Review Group, the CONSORT Group, the Scottish Clinical Trials units and the EC FP7 DECIDE project. We also have a list of researchers who have worked with PRECIS, some of whom have contacted us directly regarding collaboration.

### Informed consent

Consent will be implied by the participants if they agree to participate by returning completed questionnaires in the different phases. With participants’ permission, the Phase 2 discussion in the one-to-one or group testing will be audio recorded and then transcribed. If a participant would rather that the discussion is not recorded, we will not record the discussion and take only written notes during the discussion. Audio recordings will be stored digitally as encrypted files on University servers, protected by standard University firewall and back-up processes. Data will be kept for no longer than 5 years.

### Duration

The project started on 1 October 2012 and will run for 16 months. This work forms part of KL’s PhD work which finishes 30 September 2014.

### Ethics

The study was approved by the University of Dundee Ethics Committee, UREC 12107.

### Sample size and statistics

We will measure PRECIS-2’s inter-rater reliability using the intraclass correlation coefficient (ICC). The sample size depends on the number of raters and the number of trials these raters assess. As stated earlier, rating a trial using PRECIS takes between 10 and 20 minutes and we expect PRECIS-2 to be similar so the sample size is a compromise between precision and what we can reasonably ask raters to do. If we assume the ICC will be in the region of 0.7 (considered strong agreement) then 15 raters looking at 10, 15 or 20 trials would give precisions of 0.20, 0.17 and 0.14, respectively. We will aim to give our 15 raters between 10 and 15 trials to rate, depending on what Phase 1 tells us about the acceptable workload for raters; we will go beyond 15 if PRECIS-2 is quicker to complete than PRECIS.

For Phase 3 we will assess inter-rater reliability, and raters’ subjective global ratings of pragmatism compared to PRECIS-2 to assess convergent and face validity. We will determine if PRECIS-2 can accurately discriminate trials of varying pragmatism as judged by the subjective rating by calculating odds (discriminant validity).

Phase 4 will involve matched (by intervention) pairing of pragmatic and explanatory trials. All effect sizes will be converted to relative effect measures (for example, odds ratios or log odds) or difference measures (for example, mean difference). We will then perform a random effects meta-analysis across all trials and apply meta-regression analyses with type of trial (explanatory and pragmatic trials; binary coded) as an independent variable and overall effect as the dependent variable. The Cochrane Risk of Bias scores will be compared for the matched explanatory and pragmatic trials to assess internal validity, in particular: sequence generation, allocation sequence concealment; blinding of participants, personnel and outcome assessors; incomplete outcome data; selective outcome reporting; other potential threats to validity like publication bias. We will test the hypothesis that pragmatic trials have a greater risk of bias than trials that take a more explanatory approach.

We will also perform multivariate regression in to attempt to further explain variation in effect (for example, are topic of trial, risk of bias, sample size, degree of pragmatism and so on predictive of intervention effect size). We will apply backward step-wise regression to arrive at only significant covariates (*P* <0.05) and obtain *P* values. Goodness-of-fit for models will be assessed using Akaik’e Information Criterion (AIC), where lower values are better. If there are less than 10 trials per covariate, we will also apply permutation-based resampling on all significant covariates and extract resultant *P* values.

Statisticians from the Dundee Epidemiology and Biostatistics Unit will support all these analyses.

## Discussions

Healthcare systems and guideline producers need trials to provide data that are relevant to the clinical decisions made by patients, health professionals and policy-makers. Many trials, however, are less applicable than they could be because participants are highly selected (for example, people in an osteoporosis trial may be selected so that they have no co-morbidities even though many patients with osteoporosis do have them), outcomes that are not so important (for example, measuring bone density rather than fractures), only expert clinicians are included, or because of some other departure from usual practice conditions. These result in a great deal of resources being put into trials that are not as useful as they should be.

This study will involve a large number of trialists, methodologists and others to obtain suggestions for how the original PRECIS tool can be improved and will then validate a modified version of this tool, PRECIS-2. PRECIS-2 will help people designing trials think more carefully about the impact their decisions will have on others’ ability to use the results. It will also help trialists who do not have years of experience designing clinical trials to design trials. The original PRECIS tool is highly cited; a better tool is likely to have an even greater impact on trial design. Moreover, our links with UK trials units and international groups of trialists and methodologists mean that the tool can be expected to impact trial design internationally from an early stage. The study will also aim to end speculation as to whether pragmatic trials have a lower internal validity than more explanatory trials and, in addition, whether explanatory trials lead to higher estimates of treatment effect. The results of this project may have significant consequences for the way trials, especially regulatory trials, are designed.

## Study status

We have started recruiting to the qualitative work but not to the quantitative part of the study, the validation part.

## Competing interests

None known. ST and MZ were on the original team that designed the PRECIS tool published in 2009.

## Authors’ contributions

All authors contributed to the development of the study. KL wrote the first draft of the paper from the original grant proposal, with all authors commenting on it and subsequent drafts. All authors approved the final version.

## Supplementary Material

Additional file 1 **PRECIS – Pragmatic-Explanatory Continuum Indicator Summary **[10]**.** Click here for file
