# Supplementary figures and images for: Making clinical trials more relevant: improving and validating the PRECIS tool for matching trial design decisions to trial purpose
Source: Trials. 2013 Apr 27;14:115. doi: 10.1186/1745-6215-14-115 (PMC3748822; doi:10.1186/1745-6215-14-115)

## Additional materials 1

### PRECIS – Pragmatic-Explanatory Continuum Indicator Summary [10]

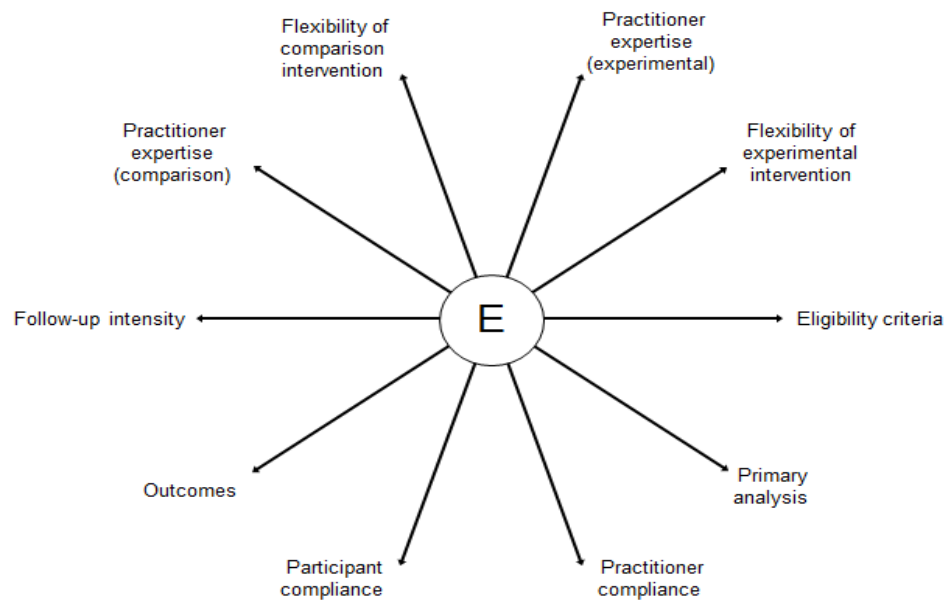

Supplement: Additional file 1 — PRECIS – Pragmatic-Explanatory Continuum Indicator Summary [10]. [file 1745-6215-14-115-S1.pdf]
